# Predictors and Outcomes of Infections in ICU Patients With Cirrhosis: A Single-Center Observational Study

**DOI:** 10.7759/cureus.47151

**Published:** 2023-10-16

**Authors:** Anand Kulkarni, Kalyan Rakam, Mahathi Avadhanam, Yogita V.P, Chinmayee Rachakonda, Anveshi Satyavadi, Asim A Zuberi, Santhosh Reddy, Sowmya Iyengar, Anand Gupta, Mithun Sharma, Nagaraja R Padaki, Reddy Rajender, Nageshwar Reddy Duvvur

**Affiliations:** 1 Hepatology, Asian Institute of Gastroenterology (AIG), Hyderabad, IND; 2 Critical Care Medicine, Asian Institute of Gastroenterology (AIG), Hyderabad, IND; 3 Clinical Pharmacy, Asian Institute of Gastroenterology (AIG), Hyderabad, IND; 4 Perelman School of Medicine, University of Pennsylvania, Philadephia, USA; 5 Gastroenterology, Asian Institute of Gastroenterology (AIG), Hyderabad, IND

**Keywords:** norfloxacin prophylaxis, sepsis-3 criteria, sofa score, fever, multidrug-resistant infections

## Abstract

Background: Patients with cirrhosis are susceptible to infections, especially by multidrug-resistant organisms (MDROs). There are limited data on the incidence of culture-positive infections and the validity of Sepsis 3-criteria in patients with cirrhosis admitted to the intensive care unit (ICU) in India, which we aimed to assess.

Methods: In this prospective study, we included consecutive patients with cirrhosis admitted to the ICU between November 1, 2021, and April 30, 2022. The primary objective was to compare the outcomes of patients with microbiologically proven infections with those without proven infections. The secondary objective was to assess the predictors of infections and mortality and the impact of drug-resistant organisms.

Results: A total of 298 patients (9.4% women) were included. The incidence of microbiologically proven infection was 34% (101/298; 95%CI=27.6-41.2). Most patients (61%) had healthcare-associated infections, Gram-negative organisms accounted for 75.3%, and bacteremia was the commonest site. Drug-resistant organisms accounted for 52.5% (53/101; 95%CI=39.3-68.7), of which 39.6% were multidrug-resistant (MDR) and 12.8% were extensively drug-resistant (XDR). Mortality was significantly higher in patients with proven infections than those without (61.4% vs. 44.2%; P=0.007). The sequential organ failure assessment (SOFA) score (OR=1.91; 95%CI=1.04-3.52; P<0.001) and presence of fever and/or positive quick SOFA (qSOFA; OR=1.91;1.04-3.52; P=0.03) were associated with an increased risk of infections. The SOFA score (OR=1.06;95%CI=1.002-1.12; P=0.04), MELD NA score (OR=1.08;95%CI=1.05-1.12; P<0.001), and presence of fever and/or positive qSOFA (OR=2.19; 95%CI=1.27-3.76; P=0.005) predicted mortality.

Conclusions: One-third of the patients with cirrhosis admitted to the ICU had microbiologically proven infection, and the mortality rate in such patients was high. SOFA, qSOFA, and fever can predict microbiologically proven infections and mortality in patients with cirrhosis.

## Introduction

Cirrhosis is a state of relative immunodeficiency characterized by hypocomplementemia, impaired clearance of bacterial products, reduced neutrophil and natural killer cell activity, and increased bacterial translocation through the leaky gut [1]. This results in increased levels of endotoxins and cytokines characterized by an exaggerated inflammatory response and hyperdynamic circulation [1,2]. This inflammatory cascade triggered by bacterial infections rapidly evolves into sepsis with multiorgan failure and acute-on-chronic liver failure (ACLF), making it a frequent trajectory and resulting in mortality within this population [3,4]. Antimicrobial resistance (AMR) is a global threat. Patients with cirrhosis are at a high risk of infection by drug-resistant organisms due to various factors, including recurrent prolonged hospitalization and invasive procedures, such as ascitic fluid drainage, variceal ligation, vascular lines, indwelling catheters, and feeding tubes [2]. Frequent contact with hospital settings can blur the well-defined boundaries between community and hospital pathogens owing to microbial circulation, which complicates epidemiology and contributes to AMR.

However, there is a paucity of data documenting AMR patterns worldwide. It is estimated that, by 2050, the impact of AMR can potentially lead to 10 million deaths [5]. The resulting economic repercussions could cost the world 100 trillion USD or a 2%-3.5% reduction in gross domestic product (GDP). Thus, if the current trends continue, AMR will drastically affect healthcare worldwide. The worldwide prevalence of multidrug-resistant (MDR) organisms (MDROs) has been rising consistently over the years, with the latest estimates nearing 40% among patients with cirrhosis [6]. Among the continents, Asia has the highest prevalence, and North America has the lowest. In the global study by Piano et al., India was reported to have the highest incidence of MDRs and extensively drug-resistant (XDR) organisms (73% and 33%, respectively) [6]. However, only a few centers from North India were included in this study, limiting the true representativeness. Furthermore, only a few studies have evaluated the impact of sequential organ failure assessment (SOFA) scores and Sepsis-3 criteria in patients with cirrhosis and infections [7,8].

In this context, our study assumes significance because infections in cirrhosis are common and are associated with poor prognosis and there are limited data comparing the outcomes of proven infections with those without proven infections. Therefore, we aimed to assess and compare the outcomes of patients with cirrhosis admitted to the intensive care unit (ICU) with proven infections with those without proven infections.

## Materials and methods

In this single-center prospective study, consecutive patients with cirrhosis admitted to the liver ICU of Asian Institute of Gastroenterology (AIG) Hospitals, Hyderabad, India, between November 1, 2021, and April 30, 2022, were assessed for inclusion. The study protocol was approved by the Institutional Ethics Committee (AIG/IEC-BH&R 20/11.2021-01) and adhered to the modified guidelines of the Declaration of Helsinki and Istanbul. Patients with human immunodeficiency virus infection (HIV) and hepatocellular carcinoma (HCC) and those who did not consent to participate in the study were excluded. The primary objective was to compare the clinical features and outcomes of patients with microbiologically proven infections with those without proven infections. The secondary objective was to assess the predictors of infection and mortality in patients with cirrhosis and the impact of drug-resistant organisms on outcomes. We followed the Strengthening the Reporting of Observational Studies in Epidemiology (STROBE) guidelines to prepare the manuscript.

Definitions

Cirrhosis was identified based on the clinical history of decompensation (ascites, variceal bleeding, and hepatic encephalopathy), imaging, and/or histological features suggestive of cirrhosis. Infections were classified as community-acquired, healthcare-associated, or nosocomial, based on the timing of symptoms of infection. Infection diagnosed within 48 hours of admission without any prior history of hospitalization for six months was labeled as community-acquired; infection diagnosed within 48 hours of admission in patients hospitalized for at least two days in the previous six months was labeled as healthcare-associated; and infection diagnosed after 48 hours of admission was labeled as nosocomial. An individual who developed a second infection separate from the first infection during the same hospitalization was labeled as a secondary infection [9]. The criteria for identifying infection sites were based on previously published standard definitions [9,10].

MDR bacteria were defined as nonsusceptibility to at least one agent in at least three antimicrobial categories or infection by methicillin-resistant *Staphylococcus aureus* (MRSA) [11]. XDR bacteria were defined as nonsusceptibility to at least one agent in all but less than two antimicrobial categories (i.e., bacterial isolates remained susceptible to only one or two antibiotic classes). Pan drug resistance (PDR) was defined as resistance to all antimicrobial agents. Resistance was established in vitro according to Clinical and Laboratory Standards Institute (CLSI) minimal inhibitory concentration breakpoints and hospital policy. Acute kidney injury (AKI) was defined per the International Club of Ascites (ICA) as an increase in serum creatinine ≥0.3 mg/dl (≥26.5 μmol/L) within 48 hours, or a percentage increase in serum creatinine ≥50% from baseline, which is known or presumed to have occurred within the prior seven days [12]. Hepatic encephalopathy was defined as cerebral dysfunction caused by hepatic insufficiency and/or portosystemic shunts that manifest as a wide spectrum of neurological or psychiatric abnormalities ranging from subclinical alterations to coma [13].

Data collection

Baseline demographic and clinical data, including age, sex, etiology of liver disease, history of norfloxacin intake, previous hospitalization, and baseline laboratory variables, including hemogram, hepatic biochemical tests, kidney function tests, and international normalized ratio (INR), were noted. Baseline pulse rate, systolic blood pressure, diastolic blood pressure, respiratory rate, and the presence or absence of fever were also recorded. The severity of the liver disease was objectively assessed using the Child-Turcotte-Pugh, model for end-stage liver disease sodium (MELD Na), sequential organ failure assessment (SOFA), and quick SOFA (qSOFA) scores. The recruited patients were categorized according to the presence or absence of culture-positive infections. Microbiological cultures and antibiotic susceptibility tests were performed according to standard international criteria. Prior to antibiotic exposure, blood, urine and ascitic fluid were sent for culture and sensitivity assessment.

Details of the infection, including site and source, microbiological cultures, leukocyte counts, and procalcitonin levels at baseline, were noted. Patients were followed up until discharge for in-hospital mortality, duration of hospital stay, and development of a second infection.

Statistical analysis

All consecutive patients who met the inclusion criteria during the study period were enrolled in the study. The collected data were entered into Microsoft Excel. The data analysis was performed using Statistical Product and Service Solutions (SPSS) (version 29; IBM Corp., NY). Continuous variables are expressed as mean and standard deviation (SD) or median and interquartile range (IQR) for parametric and non-parametric data, respectively. The categorical variables are expressed as a percentage (n) of frequency distribution. The Student's t-test for normal distribution, Mann-Whitney U test for non-parametric distribution of continuous variables, and chi-square test for categorical variables were used to compare the two groups. The predictors of infection and mortality on multivariate analysis were assessed using stepwise logistic regression analysis using univariate variables with P<0.1 and reported as odds ratio (OR). A P-value <0.05 was considered to be statistically significant.

## Results

Baseline characteristics

Three hundred and seventeen patients were admitted to the liver ICU during the study period. Seventeen patients with HCC and two with HIV were excluded, and 298 patients were included in the study. The mean age was 51.1±13.35 years, and 9.4% of the patients were females. The most common etiology of liver disease was alcohol consumption in 59.1% of patients, followed by non-alcoholic steatohepatitis (18.1%). The mean MELD Na and SOFA scores at baseline were 28.22±9.17 and 8.68±4.93, respectively, and 69% of patients were in Child Class C. More than 50% of patients had AKI and hepatic encephalopathy at admission.

Sixty-six percent (197/298; 95%CI=57.2-76) of patients did not have microbiological evidence of infection, and only 34% (101/298; 95%CI=27.6-41.2) had microbiological evidence of infection. The mean age, sex distribution, and etiology of cirrhosis were comparable between the groups. Twenty percent (n=40) of the patients in the non-infection group, and 27.7% (n=28) of the patients in the infection group were on norfloxacin prophylaxis (P=0.18). Serum procalcitonin levels were significantly higher in patients with infections. Similarly, severity scores, including MELD Na and SOFA scores, were higher in the infection group than those in the non-infection group. A higher proportion of patients with infections had AKI (62.5% vs. 47.7%; P=0.01) and hepatic encephalopathy (68.3% vs. 50.8%; P=0.004) at admission than the non-infection group (Table 1).

**Table 1:** Baseline characteristics of the patients included in the study HBV, hepatitis B virus; HCV, hepatitis C virus; NASH, non-alcoholic steatohepatitis; AIH, autoimmune hepatitis; INR, international normalized ratio; TLC, total leukocyte count; SBP, systolic blood pressure; DBP, diastolic blood pressure; MELD NA, model for end-stage liver disease sodium; SOFA, sequential organ failure assessment score; CTP, Child-Turcotte Pugh; AKI, acute kidney injury

Variables	Total (N=298)	No infection (N=197)	Infection (N=101)	P
Age (years)	51.1±13.35	50.23±13.45	52.71±13.07	0.13
Males (n, %), Females (n, %)	270 (90.6%), 28 (9.4%)	179 (91%), 18 (9%)	91 (90%), 10 (10%)	0.83
Etiology of liver disease (n,%)				
Alcohol	177 (59.4%)	115 (58.4%)	62 (61.4%)	0.44
NASH	54 (18.1%)	38 (19.3%)	16 (15.8%)	
HBV/HCV	33 (11.1%)	20 (10.2%)	13 (13%)	
AIH	5 (1.7%)	2 (1%)	3 (3%)	
Others	29 (9.7%)	22 (11.2%)	7 (7%)	
History of norfloxacin prophylaxis (n, %)	68 (22.8%)	40 (20.3%)	28 (27.7%)	0.18
Reason for ICU admission				
Hepatic encephalopathy	132 (44.3%)	90 (45.7%)	42 (41.6%)	0.29
Hypotension (MAP<65 mmHg)	37 (12.4%)	26 (13.2%)	11 (11%)	
Variceal bleed	18 (6%)	13 (6.6%)	5 (5%)	
Tense ascites	24 (8.1%)	17 (8.6%)	7 (6.9%)	
Advanced liver failure with kidney injury	33 (11.1%)	23 (11.7%)	10 (9.9%)	
≥2 reasons	52 (18.1%)	28 (14.2%)	26 (25.7%)	
Fever in past 7 days	83 (27.9%)	40 (20.3%)	43 (42.6%)	<0.001
H/o hospitalization	122 (41%)	55 (27.9%)	67 (66.3%)	<0.001
Serum bilirubin (mg/dl)	11.44±12.1	11.41±13.02	11.5±10.1	0.95
INR	2.13±1	2.06±0.99	2.27±1.02	0.09
Albumin (g/dl)	2.74±0.6	2.73±0.56	2.74±0.66	0.88
TLC (cells/mm^3^)	13,506.42±10,160.7	13,398.54±10,112.46	13,716.83±10,301.52	0.79
Serum creatinine (mg/dl)	2.24±1.8	2.1±1.68	2.53±1.98	0.04
Serum sodium (meq/dl)	131±8.1	131.08±8.04	130.89±8.24	0.84
Respiratory rate (/min)	23.87±4.62	23.15±4.38	25.28±4.77	<0.001
SBP (mmHg)	103.6±18.87	105.67±18.04	99.57±19.86	0.008
DBP (mmHg)	65.74±15.76	67.38±15.72	62.54±15.43	0.01
MELD NA	28.22±9.17	26.88±9.35	30.85±8.22	<0.001
SOFA score	8.68±4.93	6.91±3.92	12.12±4.9	<0.001
CTP Class (A/B/C)	13/79/206	11/61/125	2/18/81	0.01
AKI (n, %)	157 (52.7%)	94 (47.7%)	63 (62.5%)	0.02
Hepatic encephalopathy (n, %)	169 (56.7%)	100 (50.8%)	69 (68.3%)	0.004

Infections details

Healthcare-associated infections were the most frequent type (61%), followed by nosocomial (25%) and community-acquired infections (14%). Of the 101 patients with proven infection, drug-resistant organisms accounted for 52.5% (53/101; 95%CI=39.3-68.7). Of these, MDROs accounted for 39.6% (40/101; 95%CI=28.3-54) of infections, and XDRs accounted for 12.8% (13/101; 95%CI=6.8-22). Gram-negative organisms were the most common cause of infections noted in 76 patients (75.3% of 101). Among the 76 Gram-negative organisms isolated, 35 were carbapenem-resistant *Enterobacteriaceae*. Gram-positive organisms were isolated from 16% of patients, and fungi (*Candida albicans*) were isolated from 8%. *Klebsiella* (31.7%) and *Escherichia coli* (29.7%) were the most frequently isolated organisms. The most frequent site of culture positivity was the blood in 51.5% of patients, followed by the urine and lungs in 18%. Eight percent of the patients had proven skin and soft tissue infection and ascitic fluid infection (Table 2). Fourteen percent had more than one site positivity.

**Table 2:** Details of the infection SSTI, skin and soft tissue infection

Detail	N (%) out of 101 patients
Type of infection	
Community-acquired	14 (13.8%)
Healthcare-associated	25 (24.7%)
Hospital (nosocomial) acquired	62 (61.4%)
Site of infection	
Blood	52 (51.5%)
Urine	18 (17.8%)
Pneumonia	17 (16.8%)
SSTI	8 (8%)
Ascitic fluid	8 (8%)
Organism isolated	
Klebsiella	32 (31.7%)
Escherichia coli	30 (29.7%)
Staphylococcus	4 (4%)
Candida albicans	8 (8%)
Enterococcus	8 (8%)
Acinetobacter	4 (4%)
Others	15 (14.8%)

Twenty-two percent of patients with initial clinical evidence of infection developed secondary infections during hospitalization. The hospital stay was longer in those with infections (4.26±3.52 days) than in those without infections (3.34±2.97 days; P=0.01). Compared to patients with harboring sensitive organisms, those with resistant organisms had lower systolic and diastolic blood pressure and lower albumin levels. The most common drug-resistant organisms were *E. coli* and *Klebsiella*, and none of them was community-acquired. Details of patients' characteristics with sensitive and drug-resistant organisms are provided in Table 3.

**Table 3:** Comparison of sensitive and drug-resistant organisms HBV, hepatitis B virus; HCV, hepatitis C virus; NASH, non-alcoholic steatohepatitis; AIH, autoimmune hepatitis; INR, international normalized ratio; TLC, total leukocyte counts; SBP, systolic blood pressure; DBP, diastolic blood pressure; MELD Na, model for end-stage liver disease sodium; SOFA, sequential organ failure assessment score; CTP, Child-Turcotte Pugh; AKI, acute kidney injury

Variables	Sensitive organism (N=48)	Resistant organism (N=53)	P value
Age (years)	53.1±12.24	52.36±13.9	0.77
Females (n, %)	4 (8.3%)	6 (11.3%)	0.43
Etiology (n, %)			
Alcohol	28 (58.3%)	34 (64.2%)	0.64
NASH	6 (12.5%)	10 (19%)	
HBV/HCV	8 (16.7%)	5 (9.4%)	
AIH	2 (4.2%)	1 (2%)	
Others	4 (8.3%)	3 (5.7%)	
History of norfloxacin prophylaxis (n, %)	17 (35.4%)	11 (20.8%)	0.07
Reason for ICU admission			
Hepatic encephalopathy	22 (45.8%)	20 (37.7%)	0.37
Hypotension (MAP<65 mmHg)	4 (8.3%)	7 (13.2%)	
Variceal bleed	1 (2.1%)	4 (7.5%)	
Tense ascites	5 (10.4%)	2 (3.8%)	
Advanced liver failure with kidney injury	3 (6.3%)	7 (13.2%)	
≥2 reasons	13 (27.1%)	13 (24.5%)	
H/o hospitalization	29 (60.4%)	38 (71.7%)	0.16
Fever in past 7 days	23 (48%)	20 (37.7%)	0.2
Serum bilirubin (mg/dl)	10.5±9.88	12.4±10.32	0.34
INR	2.08±0.77	2.44±1.18	0.07
Albumin (g/dl)	2.86±0.54	2.64±0.74	0.09
TLC (cells/mm^3^)	12,291.66±10,212.66	15,007.54±10,307.57	0.18
Serum creatinine (mg/dl)	2.4±1.85	2.66±2.1	0.51
Serum sodium (meq/dl)	129.93±7.16	131.75±9.1	0.27
Respiratory rate (/min)	25.42±4.81	25.15±4.8	0.78
SBP (mmHg)	103.5±20	96±19.18	0.05
DBP (mmHg)	65.88±17.2	59.53±13.1	0.03
MELD Na	29.92±8.31	31.7±8.12	0.27
SOFA score	11.4±4.95	12.77±4.8	0.16
CTP Class (A/B/C)	2/11/35	0/7/46	0.12
AKI (n, %)	30 (62.5%)	33 (62.3%)	1
Hepatic encephalopathy (n, %)	35 (72.9%)	34 (64.2%)	0.23
Type of infection			
Community-acquired	14 (29.2%)	0	<0.001
Healthcare-associated	10 (20.8%)	15 (28.3%)	
Hospital (nosocomial) acquired	24 (50%)	38 (71.7%)	
Organism			
Klebsiella	11 (23%)	21 (37.7%)	<0.001
Escherichia coli	6 (12.5%)	24 (45.3%)	
Staphylococcus	4 (8.3%)	0	
Candida albicans	8 (16.7%)	0	
Enterococcus	6 (12.5%)	2 (3.8%)	
Acinetobacter	0	4 (7.5%)	
Others	13 (27.1%)	2 (5.5%)	

Predictors of infection

On univariate analysis, MELD Na, SOFA score, AKI at admission, history of hospitalization, presence of fever in the past seven days, and a positive qSOFA score predicted the development of infection. On multivariate stepwise logistic regression analysis, the SOFA score (OR=1.26; 95%CI=1.18-1.34; P<0.001) and history of hospitalization (OR=3.9; 95%CI=2.18-6.97; P<0.001) increased the risk of proven infections. In the second model, excluding the history of hospitalization, the SOFA score (OR=1.91; 95%CI=1.04-3.52; P<0.001) and presence of fever in the previous seven days and/or a positive qSOFA score (OR=1.91; 95%CI=1.04-3.52; P=0.03) increased the risk of proven infections (Table 4). Despite removing AKI and creatinine for collinearity, the SOFA score remained the only predictor of infection. Norfloxacin prophylaxis was not associated with an increased risk of infections (even by MDROs) (OR=1.5; 95%CI=0.86-2.62; P=0.15). Due to fewer patients, we were unable to identify any predictor of MDR infections.

**Table 4:** Univariate and multivariate predictors of infection on stepwise logistic regression analysis #excluding history of hospitalization HE, hepatic encephalopathy, MELD Na, model for end-stage liver disease sodium; SOFA, sequential organ failure assessment score; INR, international normalized ratio; AKI, acute kidney injury; TLC, total leukocyte count; qSOFA, quick SOFA

Infection	Univariate		Multivariate	P value
Age	1.01 (0.99-1.03)	0.13		
Female gender	1.1 (0.48-2.46)	0.83		
Alcohol vs. other etiology	1.13 (0.7-1.85)	0.61		
HE	1.35 (0.81-2.25)	0.24		
MELD Na	1.04 (1.02-1.07)	<0.001		
H/o norfloxacin prophylaxis	1.5 (0.86-2.62)	0.15		
SOFA score	1.28 (1.2-1.36)	<0.001	1.26 (1.18-1.34), 1.91 (1.04-3.52)#	<0.001, <0.001
INR	0.97 (0.91-1.03)	0.43		
Bilirubin	1 (0.98-1.02)	0.95		
Creatinine	1.14 (1.003-1.3)	0.04		
Albumin	1.02 (0.97-1.07)	0.35		
AKI at admission	1.81 (1.11-2.96)	0.01		
Sodium	0.98 (0.96-1.005)	0.13		
TLC	1	0.8		
Procalcitonin	1 (0.99-1)	0.64		
H/o hospitalization	5.09 (3.03-8.53)	<0.001	3.9 (2.18-6.97)	<0.001
Presence of fever in last 7 days	2.91 (1.72-4.92)	<0.001		
qSOFA positive	2.66 (1.61-4.4)	<0.001		
Either fever or qSOFA or both	3.73 (2.18-6.4)	<0.001	1.91 (1.04-3.52)#	0.03

Mortality

In-hospital mortality in the cohort was 50% (149/298; 95%CI=42.3-58.7). In-hospital mortality in patients with infections was 61.4% (62/101; 95%CI=51.17-71) compared to 44.2% (87/197; 95%CI=37.1-51.4) in those without infection (P=0.007). The most common cause of mortality was multiorgan failure in both groups. None of the patients underwent liver transplantation during their hospital stay. Mortality in patients with drug-resistant organisms was 64.2% (34/53) compared to 58.3% (28/48) in those with sensitive organisms (P=0.68).

Predictors of mortality

In univariate analysis, several variables, including severity score, markers of infection, and proven infection, increased the risk of in-hospital mortality. However, in the multivariate logistic regression analysis, SOFA score (OR=1.06; 95%CI=1.002-1.12; P=0.04), the MELD Na score (OR=1.08; 95%CI=1.05-1.12; P<0.001) and presence of fever in previous seven days and/or positive qSOFA (OR=2.19; 95%CI=1.27-3.76; P=0.005) predicted in-hospital mortality (Table 5).

**Table 5:** Univariate and multivariate predictors of mortality on stepwise logistic regression analysis HE, hepatic encephalopathy; MELD Na, model for end-stage liver disease sodium; SOFA, sequential organ failure assessment score; INR, international normalized ratio; AKI, acute kidney injury; TLC, total leukocyte count; qSOFA, quick SOFA

Mortality	Univariate	P value	Multivariate	P value
Age	0.99 (0.97-1.01)	0.54		
Female gender	1.61 (0.73-3.57)	0.23		
Alcohol vs. other etiology	1.28 (0.8-2.04)	0.28		
HE	1.41 (0.9-2.23)	0.13		
MELD Na	1.11 (1.07-1.14)	<0.001	1.08 (1.05-1.12)	<0.001
H/o norfloxacin prophylaxis	1 (0.58-1.71)	1		
SOFA score	1.15 (1.09-1.21)	<0.001	1.06 (1.002-1.12)	0.04
INR	1.06 (0.94-1.21)	0.29		
Bilirubin	1.05 (1.02-1.07)	<0.001		
Creatinine	1.21 (1.06-1.4)	0.005		
Albumin	1.03 (0.95-1.11)	0.46		
AKI at admission	2.33 (1.46-3.71)	<0.001		
Sodium	0.98 (0.96-1.008)	0.23		
TLC	1	0.002		
Proven infection	2.01 (1.23-3.27)	0.005		
Second infection	1.91 (0.67-5.4)	0.22		
H/o hospitalization	2.2 (1.37-3.52)	0.001		
Resistant organisms vs. nonresistant	1.27 (0.57-2.83)	0.54		
Presence of fever in last 7 days	3.7 (2.12-6.41)	<0.001		
qSOFA positive	3.19 (1.98-5.12)	<0.001		
Either fever or qSOFA or both	3.45 (2.13-5.6)	<0.001	2.19 (1.27-3.76)	0.005

## Discussion

The salient features of the current study are a) 34% of patients with cirrhosis in the ICU have microbiologically proven infection, b) drug-resistant organisms account for 52.5% of infections, c) the SOFA score can predict infection and mortality, d) fever and qSOFA score can predict infection and mortality, and e) norfloxacin prophylaxis is not associated with an increased risk of MDROs.

Microbiologically proven infection has been variably reported in 35-60% of patients with cirrhosis, which is similar to the results of our study [6,14,15]. The bacterial distribution of the cultured organisms was predominantly Gram-negative in our cohort. This is likely due to the translocation of Gram-negative enteric organisms into the circulation. The prevalence of MDROs varies greatly across geographical regions, but the rates worldwide are constantly on the rise. The incidence of MDROs reported in the current study was relatively lower than that reported in other centers in the northern part of India [6,16]. The reason for this can be due to strict infection control practices, stringent antibiotic policies, regular vancomycin-resistant enterococci (VRE), and MRSA screening for healthcare workers and patients. Contrary to previous studies, we noted comparable mortality rates among MDR and non-MDR infections [6,14,17]. Most infections in the MDR group were nosocomial or hospital-acquired, implying a higher antibiotic exposure or prolonged hospitalization in this group. The mortality of microbiologically proven infections was higher, even in the absence of MDROs, implying higher mortality in patients with cirrhosis and proven infection. Another reason for this may be the higher prevalence of bacteremia in the current study, which is usually associated with higher mortality [18-20].

SOFA and qSOFA scores have been validated as ideal tools for predicting mortality in patients with cirrhosis and infection [7,8]. Fever, a classical symptom of underlying infection, and qSOFA assessment can be used to make a reasonable clinical prediction for developing infection and mortality. These simple tools can be used at the bedside and are valuable for the diagnosis and prognostication of patients with cirrhosis. Several reports have explored the potential of procalcitonin as a reliable marker for correctly identifying infections and systemic inflammatory response syndrome in cirrhosis [21-23]. However, an ideal cut-off remains elusive, largely due to variations in testing techniques and diversity in study populations [2]. We did not observe any predictive association between procalcitonin and infections or mortality probably due to a higher incidence of kidney injury in our cohort, which may have influenced procalcitonin levels.

Norfloxacin prophylaxis is recommended for patients with decompensated cirrhosis and spontaneous bacterial peritonitis (SBP) with low ascitic fluid protein levels and has been reported to be effective in improving three- and 12-month survival [24-26]. There is a reported association between asymptomatic intestinal colonization with MDROs and prophylactic norfloxacin, leading to an inadvertent increase in nosocomial MDRO infections [17,27]. However, this is contentious, as four major studies conducted across the world have reported no increase in MDRO with prophylactic use of norfloxacin [6,10,14,28]. Similarly, in the current study, there was no association between norfloxacin prophylaxis and MDROs.

Limitations: The major limitation of this study is that it was a single-center study that included only those patients admitted to the ICU. While there have been no studies evaluating such homogenous populations, further multicenter studies are required to validate these findings.

Most guidelines recommend third-generation cephalosporins (TGCs) as an empirical treatment for community-acquired infections [25,29]. If current AMR trends continue, this approach may require caveats in regions where AMR is high, leading to the circulation of MDROs in the community, thus rendering the TGCs ineffective [30]. The greatest peril resulting from this scenario is inadequate MDRO coverage by currently used empirical antibiotics. This results in therapeutic failure, which translates to adverse clinical outcomes. These newer trends are likely to be dynamic; therefore, there is a need for frequent monitoring of antibiotic strategies to ensure coverage as per local epidemiological patterns for the empirical treatment of infections in patients with cirrhosis.

## Conclusions

Infections are common in patients admitted to the ICU. The current study is a significant contribution as it highlights that at least one in three patients with cirrhosis will have a microbiologically proven infection and one in six will have an infection caused by drug-resistant organisms. Further, SOFA scores, qSOFA scores, and fever can identify infections and predict mortality early. However, the outcomes of these patients with cirrhosis and infections remain poor. Further, studies should focus on the prevention of infections in these patients.
